# Genome Reduction in the Mosquito Symbiont *Asaia*

**DOI:** 10.1093/gbe/evy255

**Published:** 2018-11-23

**Authors:** Diego Peres Alonso, Maria Vittoria Mancini, Claudia Damiani, Alessia Cappelli, Irene Ricci, Marcus Vinicius Niz Alvarez, Claudio Bandi, Paulo Eduardo Martins Ribolla, Guido Favia

**Affiliations:** 1 Biotechnology Institute (IBTEC) & Biosciences Institute at Botucatu (IBB), Sao Paulo State University (UNESP), Sao Paulo, Brazil; 2 School of Biosciences and Veterinary Medicine, University of Camerino, Italy; 3 Clinical Pediatric Research Center Romeo and Enrica Invernizzi, Department of Biosciences, University of Milan, Italy; 4 MRC-University of Glasgow-Centre for Virus Research, Glasgow, United Kingdom

**Keywords:** *Anopheles darlingi*, *Asaia*, symbiosis, genome reduction

## Abstract

Symbiosis is now recognized as a driving force in evolution, a role that finds its ultimate expression in the variety of associations bonding insects with microbial symbionts. These associations have contributed to the evolutionary success of insects, with the hosts acquiring the capacity to exploit novel ecological niches, and the symbionts passing from facultative associations to obligate, mutualistic symbioses. In bacterial symbiont of insects, the transition from the free-living life style to mutualistic symbiosis often resulted in a reduction in the genome size, with the generation of the smallest bacterial genomes thus far described. Here, we show that the process of genome reduction is still occurring in *Asaia*, a group of bacterial symbionts associated with a variety of insects. Indeed, comparative genomics of *Asaia* isolated from different mosquito species revealed a substantial genome size and gene content reduction in *Asaia* from *Anopheles darlingi*, a South-American malaria vector. We thus propose *Asaia* as a novel model to study genome reduction dynamics, within a single bacterial taxon, evolving in a common biological niche.

## Introduction

Acetic acid bacteria (AAB) are obligate aerobes, belonging to various genera assigned to the family of Acetobacteraceae (Kersters et al. 2006), and the genus *Asaia* represents a particular member of AAB: It oxidizes acetate and lactate to carbon dioxide and water, but not ethanol to acetic acid (Yamada et al. 2000; Katsura et al. 2001; Yukphan et al. 2004; Malimas et al. 2008).

Several species and strains of the genus *Asaia* were firstly isolated from tropical flowers (Yamada et al. 2000; Katsura et al. 2001; Yukphan et al. 2004, 2005; Malimas et al. 2008). In particular, *Asaia bogorensis* was isolated from orchid tree and plumbago flowers, in fermented glutinous rice and in fruit-flavored bottled water (Yamada et al. 2000). Some species were reported to be human opportunistic pathogens in immunocompromised patients (Snyder et al. 2004; Tuuminen et al. 2006; Juretschko et al. 2010).

Additionally, the symbiotic mutualism between *Asaia* and arthropods was demonstrated, particularly in mosquitoes (Favia et al. 2007, 2008), encompassing *Anopheles*, *Aedes*, and *Culex* genera, well-known vectors of parasites and arboviruses causing devastating infectious diseases of public health importance, including malaria, dengue and Zika.

*Asaia* colonization within its hosts is characterized by a wide tissue tropism, being harbored in the gut, the salivary glands and the reproductive tracts of female and male individuals, corresponding to the mode of its horizontal transmission (cofeeding) within a population and its vertical transmission between host generations (parental transmission) (Damiani et al. 2008; Crotti et al. 2009; Gonella et al. 2012). Studies aimed at understanding the features of AAB symbiotic alliances with their hosts mostly focused on the investigation of possible benefits provided by AAB to their respective hosts (Ryu et al. 2008; Lee et al. 2013). Key traits of *Asaia’*s intimate interaction with the insect host include the capacity to colonize different host tissues and the ability to interact with the innate immunity and the developmental pathways of its host, as occurring also in other insect–symbiont relationships (Ryu et al. 2008; Gross et al. 2009; Douglas et al. 2011; Shin et al. 2011; Lee et al. 2013; Login and Heddi 2013).

*Asaia*, in particular, exerts a beneficial role during the development of *Anopheles stephensi* larvae (Chouaia et al. 2012), impacting also on expression of genes related the formation of cuticles (Mitraka et al. 2013).

These features led to the development of research projects aimed at exploiting *Asaia* for paratransgenic interventions against insect-borne diseases and particularly mosquito borne diseases: Its ability to easily invade and persist in populations and the efficiency in *Plasmodium* blockage when recombined with antiparasites effectors are particularly intriguing evidences for its application as a control tool (Bongio and Lampe 2015; Mancini et al. 2016).

Numerous examples of bacterial symbiosis show how variable the degree of dependence between the symbiont and its host could be: Usually this variation depends on how advantageous the associations are, and drives co-evolutionary adaptations.

Investigations on the multiple aspects of mutualistic relationships, undeniably involves the analysis of the nature of the microbial acquisition and of the co-evolutionary processes between the symbiont and its host.

Surprisingly, evolutionary reconstructions of various microbial symbiotic associations revealed that genome simplification often occurs, resulting in the loss or lack of genes that are essential in other related bacteria. Particularly, genome reduction is widely spread among maternally inherited symbiotic microbes, being a common strategy to reduce the cost of genome replication, as the result of the co-evolution between the symbiont and its host (Tian et al. 2017). Sloan and Moran (2012) recently pointed out that the most extreme examples of bacterial genome reduction are those reported in insect endosymbionts providing essential nutrients to their hosts.

Through a de novo genome sequencing and comparison of *Asaia* isolates from various mosquito species we detected substantial variations in the genomic architecture of the strain isolated from field-collected *Anopheles darlingi*, the main South-American malaria vector. In particular, we discovered that its genome is reduced in comparison with other isolates from mosquito species belonging to *Anopheles* and *Aedes* genera. Therefore, *Asaia*, similarly to *Serratia symbiotica* (Manzano-Marín and Latorre 2016), is proposed as a good model to study the genome reduction dynamics within a single bacterial taxon evolving in the mosquito host, representing a common biological niche.

## Materials and Methods

### *Asaia* Strains Isolation from Adult Mosquitoes

*Asaia* strains were newly isolated from *An. darlingi* (*Asaia ADar*) and *An. funestus* (*Asaia AFun*). Adult females of *An. darlingi* were collected by human landing in the area of the village of Mancio Lima, Acre State, Brazil; the sampling site (7°37′14S; 72°53′7W) include two large detached dwellings with annex large artificial fish ponds, bordering with the Amazon forest. Genomic DNA was extracted using Wizard Genomic DNA Purification Kit (Promega, WI).

Mosquito species identification of adults was achieved by the amplification of a fragment of the mitochondrial cytochrome c oxidase subunit I gene (COI) using LCO 1490-HCO 2198 primers pair (Folmer et al. 1994). Amplicons were sequenced and analyzed by BlastN.

*Asaia* AFun was isolated in 2008, from *An. funestus* females collected by indoor resting, near the village of Kuiti (Ouagadougou, Burkina Faso) (12°11′36″N; 1°23′11″W). Mosquito species were assessed by morphological identification, using recognition keys.

The same protocol was used for all isolations from mosquito adults.

Freshly collected mosquitoes were anesthetized and surface sterilized by immersion in 75% ethanol and washed twice in PBS. *Asaia* was isolated by grinding whole mosquitoes in a pre-enrichment liquid medium (2% sorbitol, 0.5% peptone, 0.3% yeast extract and 100 ppm cycloheximide, with adjusted pH 3.5 with hydrochloric acid); grown bacteria were plated on a CaCO_3_ agar plate containing 2% glucose, 0.5% ethanol, 0.8% yeast extract, 0.7% CaCO_6_, and 1.2% agar: Colonies causing dissolution haloes were selected. Identification was confirmed by 16S rDNA gene analysis, using 20F-1500R primers (Yamada et al. 2000); amplicon sequencing revealed that the strain isolated from *An. darlingi* was 99% homologous with *Asaia krungthepensis* (GenBank: AB102953.2), *Asaia AFun* sequences gave 100% identity with the isolate from *An. gambiae* (GenBank: FN821396.1) (Yukphan et al. 2004; Damiani et al. 2010).

Strains isolated from *An. stephensi* (*Asaia ASte*), *An. gambiae* (*Asaia AGam*), *Ae. aegypti* (*Asaia AAeg*), and *Ae. albopictus* (*Asaia AAlb*) were previously described in several studies (Favia et al. 2007; Crotti et al. 2009; Damiani et al. 2010; Rossi et al. 2015).

### Whole-genome Sequence of *Asaia* Strains, Genomic Comparison, Gene Content, and Sequence Analysis

Total genomic DNA of all isolated *Asaia* strains was extracted with JetFlex Genomic DNA Purification kit (Genomed, Lohne, Germany) to construct a Nextera XT DNA library, which was sequenced using Next Seq platform (Illumina Inc., San Diego, CA). De novo assembly was performed using the A5-assembly pipeline and Velvet V.1.2.1 pipeline. myRAST was used to predict open reading frames and BLAST (version 2.0) software was utilized to confirm the predictions. Comparative genome analysis was performed with CLC genomics workbench 7.0 using *A. bogorensis* as the reference genome. A pairwise genome comparison genetic similarity matrix containing all newly isolated *Asaia* strains and the reference genome from *A. bogorensis* was built as follows. First, based on gene annotation, only shared genes were selected for further processing. Intersected genomic sequences were then aligned using Clustal Omega. On the basis of this alignment, a matrix containing mean values of the proportion of polymorphic sites found for each gene for each pairwise genome comparison was built. Ribosomal 16S sequences (1,414 base pairs) were aligned using ClustalW 2.0.12 (Larkin et al. 2007) and phylogenetic analysis was performed with MEGA version 4 (Tamura et al. 2007). Principal component analysis (PCA), data visualization and plotting were performed in R using *ggplot2* package. Numbers of unique and mutual genes among *Asaia* isolates are summarized in a Venn diagram generated using InteractiVenn (Heberle et al. 2015).

### Motility Test

Bacterial motility test was conducted using the hanging drop procedure. Each *Asaia* strain was grown on GLY agar (yeast extract 1%, glycerol 2.5%, agar 2%, pH 5) for 48 h at 30 °C. After picking a single colony from each plate, bacteria were resuspended in a drop of 1× PBS and then observed under 100× optical microscopy. Cell motility was assessed observing multiple areas of the same slide within three biological replicates.

## Results

### Features of Genomes of Various *Asaia* Strains and Comparative Genomic Analysis

In this study, we report the successful de novo sequencing of different *Asaia* strains isolated from six *Culicidae* mosquitoes of major medical importance: *An. funestus* (*AFun* isolate); *An. gambiae* (*AGam* isolate); *An. stephensi* (*ASte* isolate); *An. darlingi* (*ADar* isolate); *Ae. aegypti* (*AAeg* isolate); *Ae. albopictus* (*AAlb* isolate).

The whole genome sequences of all *Asaia* isolates produced high quality paired-end reads ranging from 15,055,660 to 64,198,344 with a median coverage ranging from 423× to 2,151×. The draft genomes presented contig contents varying from 21 to 221, with a N50 ranging from 139,464 to 512,361 bp with a maximum contig length varying from 297,776 to 1,067,154 bp and the GC content ranged from 59.7% to 61%. The genome size varied from 3,082,691 to 3,799,680 bp. The total open reading frames predicted ranged from 2,909 to 3,718. Summarized results are presented in table 1; gene annotations for all isolates are presented as a supplementary table (supplementary table S1, Supplementary Material online).

**Table 1:** Genomic Assembly Metrics for *Asaia* Isolates

ID	Mosquito Species	Total Reads	Median Coverage	Contigs	N50	Maximum Contig Length (bp)	G + C	Genome Size (bp)	ORFs
*Asaia AAeg*	*Ae. aegypti*	26,393,932	475×	50	278,143 bp	563,029	59.7%	3,547,954	3,464
*Asaia AAlb*	*Ae. albopictus*	15,055,660	423×	89	323,414 bp	743,670	60.3%	3,799,680	3,718
*Asaia ADar*	*An. darlingi*	19,465,092	796×	21	512,361 bp	630,723	61%	3,082,691	2,909
*Asaia AFun*	*An. funestus*	30,128,800	513×	221	139,464 bp	297,776	60,2%	3,622,158	3,508
*Asaia AGam*	*An. gambiae*	64,198,344	2,151×	177	441,965 bp	1,067,154	60,3%	3,736,272	3,604
*Asaia ASte*	*An. stephensi*	19,399,854	695×	41	397,238 bp	563,044	59,7%	3,526,892	3,431

Note.—The table shows samples ID, the mosquito host from where bacteria were isolated and the sequencing metrics.

**Fig. 1.**
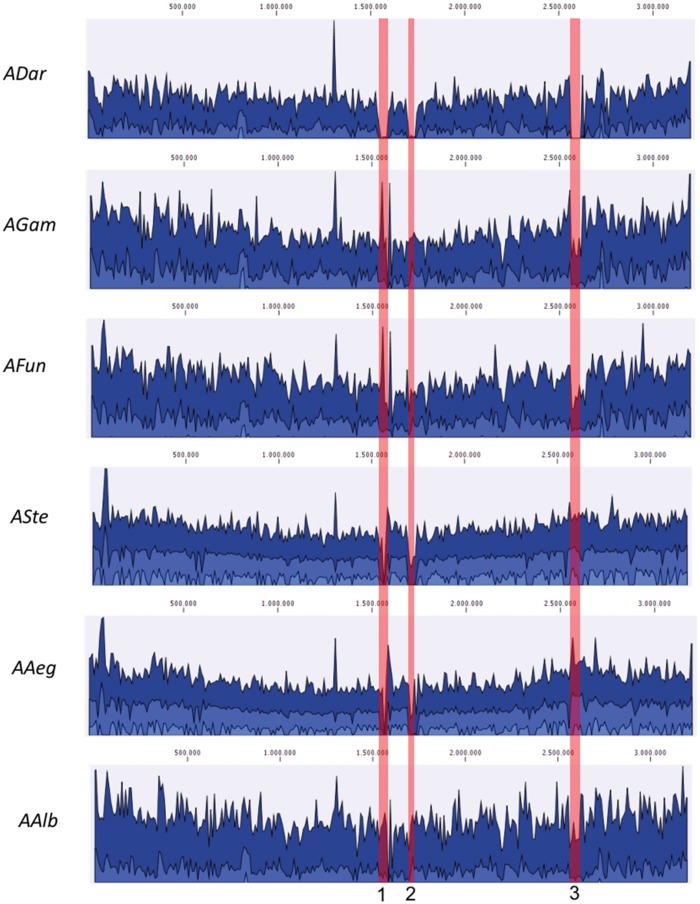
—Genome alignment comparison between *Asaia* isolates. Red bars highlight gap regions in the genome of *ADar* isolate.

Comparative genome analysis using *A. bogorensis* as the reference genome showed a homogeneous coverage among all sequenced isolates, suggesting strong synteny and close relatedness between them, with the exception of *ADar* isolate, which presented a pronounced lack of a genomic regions, highlighted in figure 1, when compared with other isolates, suggesting gene loss events.

**Fig. 2.**
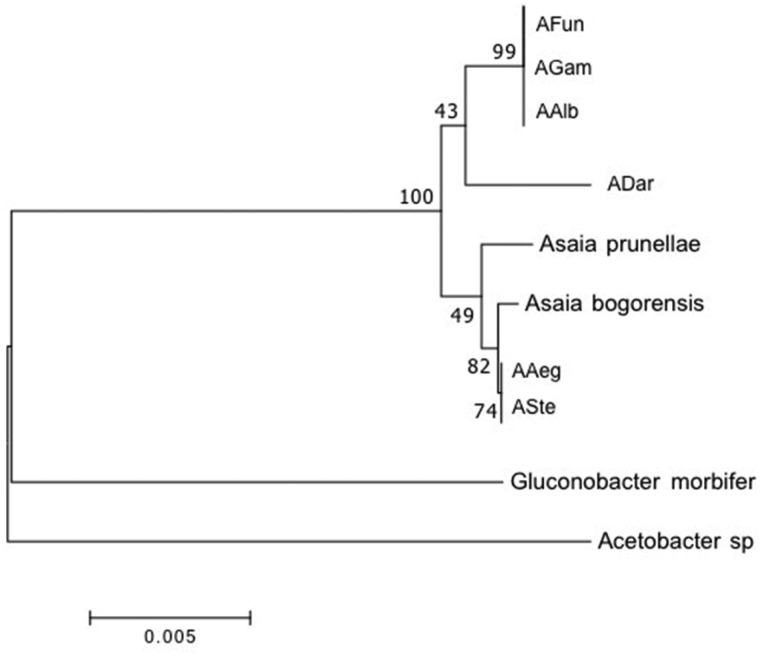
—Phylogenetic relationships of *Asaia* isolates: bootstrapped neighbor joining phylogenetic tree built on the 16S rDNA of bacterial isolates from mosquito species. Additionally, 16S rDNA sequences from two environmental species of *Asaia* (*A. bogorensis* and *A. prunellae*), and acetic acid bacteria, *Gluconobacter morbifer* and *Acetobacter* sp., are inferred from literature and used for comparison. The bar represents distance percentages.

On the basis of the 16S rDNA of all sequenced isolates, two *Asaia* species retrieved in GenBank (*A. bogorensis* and *A. prunellae*) and two members of AAB bacteria (*Gluconobacter morbifer* and *Acetobacter* sp.) closely related to *Asaia* genus used as outgroup, a phylogenetic neighbor joining (NJ) was reconstructed with bootstrap resampling (1,000 replications) (fig. 2). Interestingly, *Asaia* isolated from laboratory mosquito strains (*AAeg* and *ASte*) clustered together and are more related to *A. bogorensis* and *A. prunellae.* Moreover, in the other major branch of the tree, samples isolated from old world field collected mosquitoes (*AAlb*, *AFun*, and *AGam*) also showed a clustering pattern. The *ADar* isolate resulted completely separated from all other isolates (fig. 2). A consistent result was achieved when a maximum likelihood tree was built (supplementary fig. S1, Supplementary Material online).

**Fig. 3.**
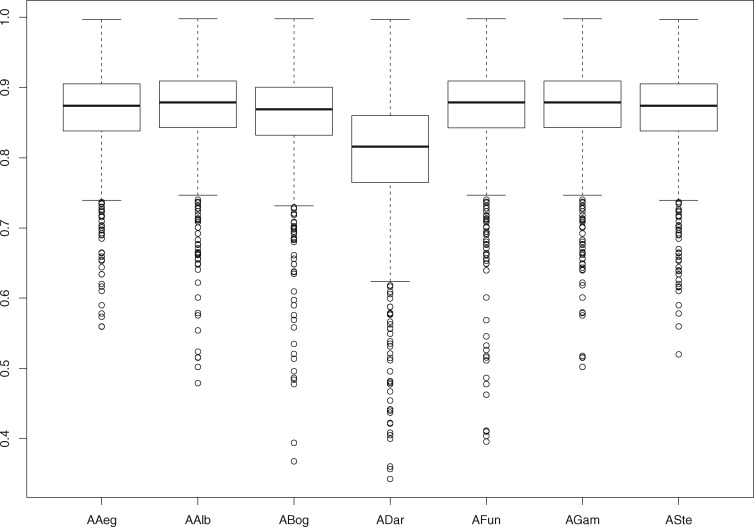
—Boxplots representing mean values of the proportion of the polymorphic sites for each gene for each isolate in comparison with others.

**Fig. 4.**
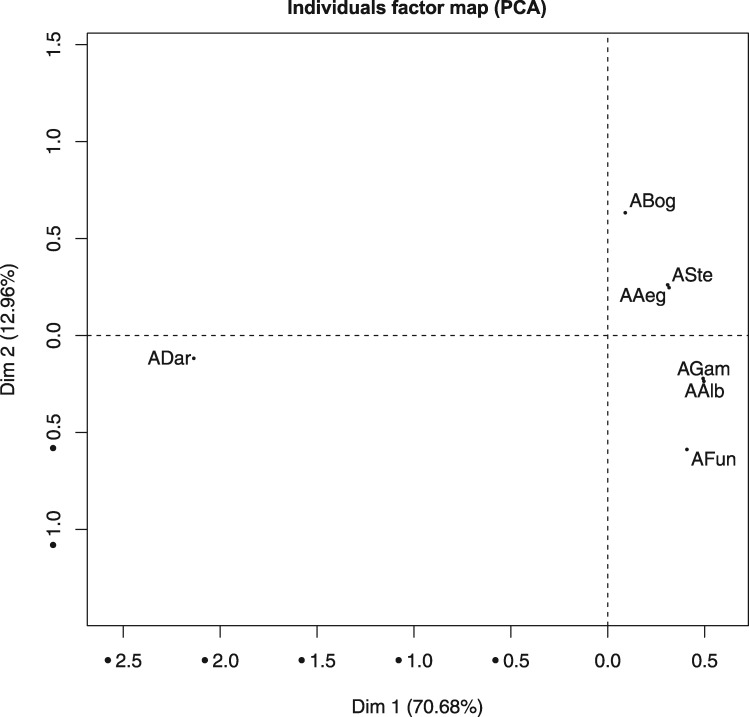
—Principal component analysis (PCA) built using shared genes from each *Asaia* isolates. *Asaia bogorensis*, an environmental isolate, is also included in the comparison.

**Fig. 5.**
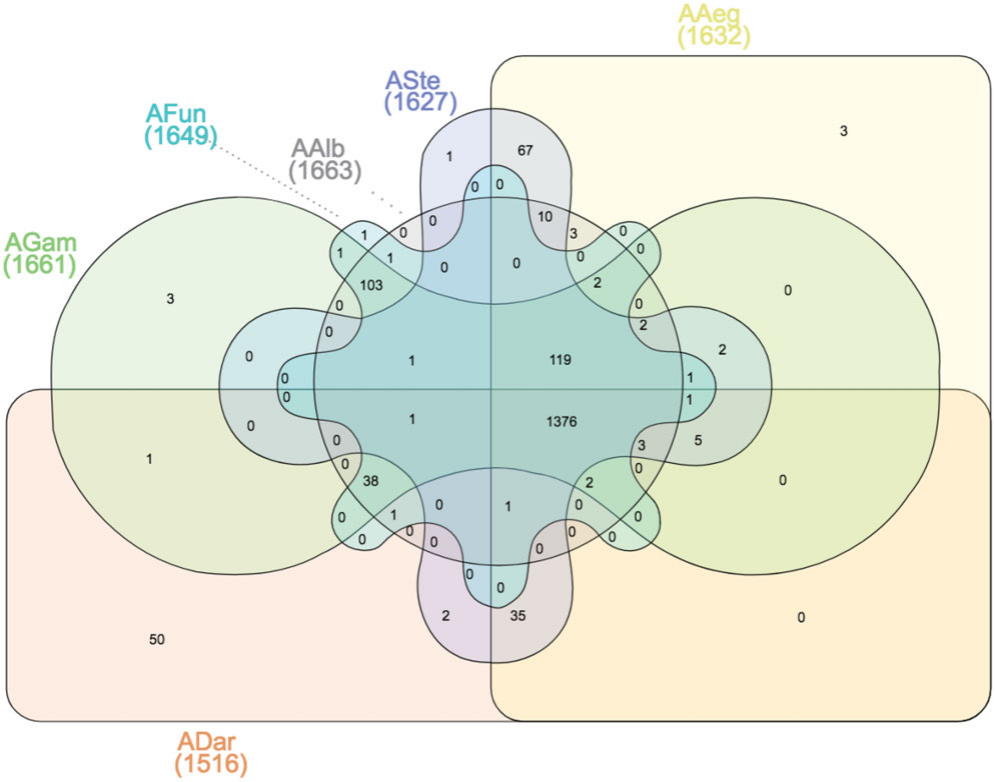
—Venn diagram summarizing the number of unique and mutual genes among isolates. The overlapping numbers specify shared genes between comparisons and nonoverlapping numbers stands for exclusive genes for each isolate. Numbers in brackets represent the total number of annotated genes per isolate.

Overall pairwise genome comparison based on polymorphic regions of all annotated shared genes by all sequenced isolates and *A. bogorensis* was performed, and a genetic similarity matrix was generated (supplementary table S2, Supplementary Material online). Interestingly, when the mean values of the proportion of the polymorphic sites for each gene for each isolate compared with all others are considered, we observed a remarkable difference in genetic similarity of *ADar* compared with all other isolates, and also to *A. bogorensis* (fig. 3). To further disclose this genetic difference in coding genomic regions in *ADar* isolate, we built a genetic similarity matrix to perform a PCA depicted in figure 4. From the PCA analysis, *ADar* isolate is strikingly different from all the other isolates with the first dimension of the PCA explaining the 71% of the overall difference. All other isolates tend to cluster together, with the second dimension explaining only around 13% of the overall difference. Interestingly, the laboratory isolates (*AAeg* and *ASte*) clustered very close to each other, although separated from the old-world field isolates (*AAlb*, *AFun*, and *AGam).* A Venn diagram (fig. 5) summarizes the number of shared genes among isolates, pinpointing that: *ADar* presents 50 unique genes, *AGam* and *AAeg* 3, whereas *AFun* and *ASte* only 1. *AAlb* does not possess any exclusive genes, but shares 1,376 genes with the other isolates, 119 when *ADar* is ignored. Interestingly, *Asaia* strains directly isolated from field mosquitoes (*AGam*, *AFun*, and *AAlb*) have 103 genes in common; when *ADar* is included 38 genes resulted to be shared. Similarly, isolates from mosquito colonies reared (*AAeg* and *ASte*) in laboratory conditions have 67 mutual genes.

**Table 2:** Number of Total Genes in Each Isolate

Isolate	Total Genes	Flagellum Complex	Mobile Elements
*AAeg*	3,464	30	25
*AAlb*	3,718	30	34
*ADar*	2,909	6	7
*AFun*	3,508	30	24
*AGam*	3,604	30	26
*ASte*	3,431	30	23

Note.—the table compares the number of total genes identified in each isolate. Specific annotated genes belonging to the flagellum machinery and mobile elements were also included.

Observing gene annotations of isolates, the major family of genes lacking in the *ADar* isolate was related to the flagellum assembly machinery and to mobile elements (table 2). Interestingly, the specific genomic region of all *Asaia* isolates containing flagellum-related genes is missing in *ADar* isolate: this region corresponds to the third highlighted red bar in figure 1.

## Discussion

From the study of the first five complete genomes of primary obligate endosymbionts of insects (belonging to *Buchnera*, *Wigglesworthia*, and *Blochmania* genera), together with many other examples, gene loss events mainly target cell membrane proteins, DNA repair and recombination genes. *ADar*, among 326, lacks 18 genes encoding cell membrane proteins or hypothetical/predictable/probable proteins, 3 genes involved in DNA recombination/repair and 10 genes composing the Trb operon, responsible for mating pair formation during conjugative transfer.

Moreover, genes encoding for ureases proteins and urea ABC transporters (7 and 5 genes respectively) are absent. In particular, bacterial ureases were recognized as important virulence factors of diseases by urease-producing microorganisms. Microbial ureases have fungitoxic properties and some bacterial ureases possess strong insecticidal effects (Carlini and Ligabue-Braun 2016). During the transition from infection to symbiosis, gene coding for virulence factors tend to be lost rapidly, as reported in *Spiroplasma*, where the pathogenic species *S. culicicola* and *S. taiwanense* share a copy of the gene encoding for the *L*-α-glycerophosphate oxidase (*glpO*), considered a virulence factor, along with a series of genes coding for related transporters (Chang et al. 2014). Interestingly mosquito commensals like *S. diminutum* and *S. sabaudiense* lack these genes; although it is worth mentioning that *glpO* is conserved in the commensal species *S. chrysopicola* and *S. syrphidicola* from deer flies and syrphid flies (Bolaños et al. 2015). In this context, other genes encoding for virulence factors are absent in *ADar*, such as those encoding for the subtilisin-like serine protease and the NAD-dependent epimerase/dehydratase.

Events of genomic reduction in symbiotic bacteria often include mobile elements, especially in endosymbionts (Latorre and Manzano-Marín 2017). When the number of mobile elements between isolates is compared, *ADar* displays the lowest number among them, 1/5 of *AAlb* and ∼1/4 of *AAeg*, *AGam*, *ASte*, and *AFun* (table 2).

Restriction and modification processes are used by bacteria to protect themselves from cellular invasion by exogenous DNA, as for example during bacteriophage infections. Interestingly, members of Type I restriction–modification machines are lacking in *ADar*: Four genes encoding for different subunits of this restriction modification system are lost. In two *genomovars* of symbionts of the genus *Elusimicrobia*, an obligate intracellular symbiont of the cellulolytic protist genus *Trichonympha* in the gut of termite *Reticulitermes speratus*, the repertoire of intact restriction–modification systems is different from others *genomovar* (Izawa et al. 2016).

The major group of genes undergoing a remarkable reduction are those associated to the flagella machinery (i.e., flagella biosynthesis proteins, flagella basal-body rod proteins, flagella motor rotation proteins) (table 2). This is not surprising since it is well documented that endosymbiotic bacteria of insects are nonmotile and their biochemical processes are intimately related to those of their host, resulting is a partial or total reduction of flagella, especially in intracellular endosymbionts (Toft and Fares 2008). This phenomenon is largely described in γ-Proteobacteria by using comparative genomic analyses: In fact, flagella genes were differentially missing in endosymbiotic bacteria of insects, suggesting a functional divergence, including a development and specialization in the export of proteins from the bacterium to the insect (Toft and Fares 2008).

The investigation of *ADar* absence of flagella genes has been expanded by using the hanging drop method, correlating genotypic and phenotypic evidences. It confirms that the cells of *ADar* isolate do not display a motile behavior, appearing static during microscope observations, when compared with other isolates.

Many other lacking genes encode for enzymatic factors: this streamlining might be explained hypothesizing that such particular enzymatic functions are no longer needed, suggesting an increasingly intimate association between *Asaia* and *An. darlingi*.

In addition to the observed genome reduction (327 genes in total), *ADar* gained 49 genes, uniquely detected in this isolate when compared with others.

These genes are mostly involved in metabolic processes, including nitrogen fixation. The most likely way of acquisition is through the bacterial–bacterial horizontal gene transfer (HGT). In fact, bacterial–bacterial HGT frequently involves genes encoding metabolic enzymes (Ochman et al. 2000). Moreover, most of these genes have not been described in insects, suggesting that the host is not the source of the acquisition. It is possible to hypothesize that these genes were acquired from some components of the bacterial network present in the gut or in other tissues of *An. darlingi*.

Four genes encode for members of the capsular polysaccharide export system (WcbC, KpsE, KpsS, and KpsC): mutations of these genes in *Rizhobium* sp. NGR234, a nitrogen fixation bacteria (like *Asaia* sp.), resulted in a defective symbiotic association with its plant hosts (Le Quéré et al. 2006). Similarly, *ADar* specifically also contains a group of genes involved in sulfate and thiosulfate transport system (CysA, CysW, CysT, and CysP), reported to be crucial for various microorganisms involved in metabolism of nitrogen, carbon and sulfur.

Another intriguing example of acquired genes is represented by the TonB-dependent hemin: This transporter has been characterized in *Sodalis glossinidius*, a *Glossina* symbiont (Hrusa et al. 2015), with important implication in the maintenance of symbiont homeostasis.

It is noteworthy that besides gene acquisition and gene loss, all coding genes in *ADar* isolate are significantly more diverse when compared with all other sequenced isolates, and to *A. bogorensis* (fig. 3). In our view, this could be the result of evolutionary pressure in coding genes related to a transition to the endosymbiotic state of the *ADar* isolate. This reasoning is further supported when the genetic divergence observed in the 16S rDNA phylogenetic tree (fig. 2), reflecting the molecular clock of the *Asaia* genera, is compared with the genetic divergence displayed in the PCA analysis for all coding genes (fig. 4), where *ADar* isolate is much more diverse from all other isolates. Moreover, from the list of all annotated genes used to build the genetic similarity matrix for the *ADar* isolate (supplementary table S2, Supplementary Material online), the less diverse genes compared with all other isolates are ribosomal proteins coding genes and the more diverse are surface proteins related genes.

In conclusion, the genome reduction of *Asaia* in *An. darlingi* could be due to a longer and more intense evolutionary relationship between symbiont and mosquito host that characterize this association with respect to those established with other mosquito species. Although our observations offer solid evidences of a specific genetic streamlining within the same symbiotic species, we are not able at this point to further characterize how this genetic diversity impacts on the symbiotic relationship with the host, and possibly on biological traits of the latter.

To the best of our knowledge, only one free-mating and long-standing *An. darlingi* colony has been successfully established in a laboratory, indicating the challenging nature of carrying out manipulation experiments.

In literature, two unique examples of bacteria undergoing genome reduction and massive gene rearrangements within the same lineage are *Coxiella* and *Serratia*.

Genome comparison between a *Coxiella*-like symbiont from *Rhipicephalus turanicus* ticks (CRt), a 2.5 times smaller genome of *Coxiella* from the lone star tick *Amblyomma americanum* (CLEAA) and other genomes from the genus *Coxiella*, such as *C. burnetii*, offers a unique picture of different stages of independent transitions to obligate tick symbionts: The analysis revealed various patterns of genome reduction, in terms of variable genome sizes and presence of common genetic marks (Gottlieb et al. 2015). For example, the well-studied *C. burnetii* showed features of ongoing genome erosion, including high number of pseudogenes and expansion of repetitive and mobile elements, suggesting being in an early phase of the host-association. When instead CRt is compared with CLEAA, a noticeable difference in genome size and in consequent alteration of metabolic and basic cellular functions is observed. We propose that a similar transition process is undergoing in the *ADar* isolate: the loss of several flagellar genes and mobile elements represents an indication of genetic marks of an early stage transition to an obligate symbiotic relationship. The complete erosion of genes responsible for the formation of the flagellar complex is a clear example of impaired cellular functions of *ADar*. Similarly to *Coxiella*, we hypothesize that an independent evolution rate is taking place rather that a longer association within the host as suggested by the longer branch of the *ADar* isolate in the phylogenetic analyses.

Interestingly, the rate of molecular evolution must be considered as a life-history trait, being affected by several factors, including species body size, population dynamics, lifestyle, and locations of the hosts (Bromham 2009). In our case, the biology, the habitat and the geographic distribution of the host (*An. darlingi*) are crucial differences between the mosquito species involved in this analysis.

Another precious reference for genome reduction in endosymbiontic systems is the analyses of lineages of *Serratia*. Sequencing of genomes of *S. symbiotica* SCc, an obligate endosymbiont strain of *Cinara cedri*, of SAp, a facultative endosymbiont of the aphid *Acyrthosiphon pisum*, and the genome of SCt, an obligate endosymbiont of the aphid *Cinara tujafilina*, elucidated the adjustments during genome reduction processes, offering evidences that differences in genome size and the rate of gene decay between the three lineages can be mainly addressed to the nature of the symbiotic association itself (obligate vs. facultative) (Manzano-Marín and Latorre 2014). It is interesting that the metrics of ADar genome size (*ADar*, 2.9 Mb and SAp, 2.79 Mb) are comparable to the facultative endosymbiotic strain of *Serratia*, and perhaps offering a snapshot of the *ADar* stage of transition pointing toward a closer symbiosis, if compared with other isolates.

Thus, our findings suggest that *Asaia*, together with other rare examples, such as *Coxiella* and *Serratia*, provide a unique opportunity to shed light on the genome-reduction phenomena not only within a single bacterial lineage, but also between isolates symbiotically associated with highly related hosts.

## Supplementary Material

Supplementary data are available at *Genome Biology and Evolution* online.

## Supplementary Material

Supplementary Data
