# Supplementary material for: Genome Reduction in the Mosquito Symbiont Asaia
Source: Genome Biol Evol. 2018 Nov 23;11(1):1–10. doi: 10.1093/gbe/evy255 (PMC6317953; doi:10.1093/gbe/evy255)
Supplement: Supplementary Data [file evy255_supp.zip › Supporting Information Legends.pdf]

Supplemental Table S1: Gene annotation from six *Asaia* isolates. Sequences were subjected to automated annotation using MyRAST. Annotations were double-screened against the non-redundant protein databases in NCBI using BLAST.

Supplemental Table S2: Overall pairwise genome comparison based on polymorphic regions of annotated shared genes between isolates and *A. bogorensis*. Intersected genomic sequences from shared genes are aligned using Clustal Omega and the mean values of the proportion of polymorphic sites found for each gene for each pairwise genome comparison are calculated.

Supplemental Fig. S1: Phylogenetic relationships of *Asaia* isolates: maximum likelihood built on the 16S rDNA of bacterial isolates from mosquito species. Additionally, two environmental species of *Asaia* (*A. bogorensi*s and *A. prunellae*), and acetic acid bacteria, *Gluconobacter morbifer* and *Acetobacter sp.*, are included and used for comparison. The bar represents distance percentages.
