# Supplementary figures and images for: Genome Reduction in the Mosquito Symbiont Asaia
Source: Genome Biol Evol. 2018 Nov 23;11(1):1–10. doi: 10.1093/gbe/evy255 (PMC6317953; doi:10.1093/gbe/evy255)

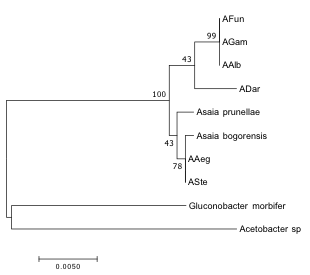

Supplement: Supplementary Data [file evy255_supp.zip › Supplementary Figure 1.docx]
